# Death and ADL Dependency After Scoring Zero on the NIHSS: A Swedish Retrospective Registry-Based Study

**DOI:** 10.1212/CPJ.0000000000200186

**Published:** 2023-09-01

**Authors:** David Darehed, Malin Reinholdsson, Adam Viktorisson, Tamar Abzhandadze, Katharina S. Sunnerhagen

**Affiliations:** Department of Public Health and Clinical Medicine (DD), Sunderby Research Unit, Umeå University; Rehabilitation Medicine Research Group (DD, MR, AV, TA, KSS), Department of Clinical Neuroscience, Institute of Neuroscience and Physiology, University of Gothenburg, and Sahlgrenska University Hospital; Department of Occupational Therapy and Physiotherapy (MR, TA); and Rehabilitation Medicine (KSS), Neurocare, Sahlgrenska University Hospital, Gothenburg, Sweden.

## Abstract

**Background and Objectives:**

Of all strokes, mild strokes (defined as 5 points or less on the National Institutes of Health Stroke Scale [NIHSS]) are in the majority. However, up to one-third of patients with mild strokes still exhibit significant deficits 3 months after the stroke. Studies on the presumably mildest strokes, defined by zero points on the NIHSS (0-NIHSS) at admission, are scarce. Hence, we aimed to study patient characteristics and outcomes among patients with 0-NIHSS strokes.

**Methods:**

Our retrospective registry-based study included a total of 6,491 adult patients with stroke admitted to 3 different stroke units in Gothenburg, Sweden, from November 2014 to June 2019. Our main outcome was a composite measure including death and activities of daily living (ADL) dependency 3 months after the stroke. Analyses of patient characteristics were followed by adjusted analyses including multiple confounders.

**Results:**

In total, 5,945 patients had data on NIHSS at admission, of whom 1,412 (24%) presented with a 0-NIHSS stroke. Among these, the median age was 72 years, 600 (42%) were female, and 86 (6%) had a hemorrhagic stroke. Among previously ADL-independent patients, 65 (6%) were either dead or ADL-dependent 3 months after the stroke. Prestroke physical inactivity (OR 2.48, 95% CI 1.40–4.38) and age (OR 1.05 per gained year, 95% CI 1.02–1.08) significantly increased the risk of death and ADL dependency.

**Discussion:**

One of 17 patients has either died or become ADL-dependent 3 months after a 0-NIHSS stroke, stressing that these strokes are not always benign. Older and physically inactive patients are at greater risk of an adverse outcome.

## Introduction

The National Institutes of Health Stroke Scale (NIHSS) has proven to be a good predictor of outcomes after stroke, where lower points are associated with more favorable outcomes.^1^ However, a caveat of the scale is that it is weighted toward language and gross motor function, while being less sensitive for brainstem and cerebellar strokes as well as cognitive functions.^2,3^ This bias is also supported by studies showing that the NIHSS underestimates infarct size in posterior circulation and right hemisphere strokes.^4,5^

Previous studies have shown that up to one-third of all patients with “mild strokes” (no universal definition exists in this article, defined as 5 points or less on the NIHSS) at presentation still exhibit significant deficits 3 months after the stroke.^6-8^ Of all strokes, mild strokes are in the majority with proportions up to two-thirds of all strokes.^9,10^ The mildest among mild strokes are those with an immeasurable neurologic deficit, defined as zero points on the NIHSS (0-NIHSS) at hospital admission. Studies focusing on 0-NIHSS strokes are scarce, and one reason for this could be that these strokes occasionally are considered as transient ischemic attacks or simply have been omitted from previous studies.^11,12^

Previous studies on patients with 0-NIHSS strokes have had study samples ranging from 12 to 108 patients.^13-16^ The largest among these studies found that 47% had a modified Rankin Scale (mRS) score of 1 or more and 29% had a mRS score of 2 or more 3 months after a 0-NIHSS stroke.^13^ The other studies found varying degrees of cognitive and motor impairment, suggesting that these strokes are not entirely harmless.^14-16^ Owing to the previous studies relatively small sample sizes on these presumably benign strokes, we aimed to use registry data to further characterize this group of patients, study outcomes, and investigate factors associated with death and ADL dependency at 3 months.

## Methods

### Design and Setting

We did this retrospective study on data from a local register on stroke care in Gothenburg (the Väststroke register), combined with data from the national Swedish Stroke Register (Riksstroke), Statistics Sweden, and the Swedish cause of death register. We included all adult (18 years or older) patients registered with a stroke (*International Classification of Diseases* 10th revision [ICD-10] code I61 [hemorrhagic stroke], I63 [ischemic stroke], or I64 [undefined stroke]) in the Väststroke register between November 2014 and June 2019. Patients with missing data on NIHSS at admission were excluded. Stroke is a clinical diagnosis in Sweden, meaning that if the patient has symptoms of a stroke lasting more than 24 hours (symptoms lasting less than 24 hours is considered a transient ischemic attack) and stroke is the most probable cause (i.e., not suspecting any stroke mimics), the patient will get a stroke diagnosis.

Gothenburg is Sweden's second largest city, with a little more than 1 million inhabitants in the metropolitan area. Patients having an acute stroke in Gothenburg are cared for at 1 hospital with 3 sites: Sahlgrenska University Hospital, Mölndal Hospital, and Östra Hospital. Neurosurgical care and reperfusion therapy are only available at Sahlgrenska, while all 3 sites have comprehensive stroke units. The number of stroke unit beds during the studied period varied, with a maximum bed availability of 68 in 2018 or ∼7 beds per 100,000 inhabitants.^17^ Finally, about one-third of all stroke patients in Sweden arrive at the hospital within 3 hours from symptoms, and more than 90% of all stroke patients admitted to hospitals are treated at stroke units, meaning that patients with even the mildest symptoms receive stroke care by a multidisciplinary team.

### Data Sources

Riksstroke is a nationwide hospital-based quality register on stroke care. All Swedish hospitals treating patients with acute stroke report to the register. In this study, data on acute care episodes as well as follow-up questionnaires at 3 months were used, including patient characteristics, care process measures, temporal data, and outcomes.^10^

The Väststroke register is a local quality register initiated in 2014, which collects data from the stroke units at the 3 abovementioned hospital sites in Gothenburg. The data collected in the register complements data from Riksstroke, including more detailed data on functional status (cognitive tests, motor function, etc.), care processes (nutritional screening, cognitive screening, etc), and outcomes (functional outcomes, quality of life, etc).^18^

Statistics Sweden is a government agency that is responsible for all kinds of official statistics in Sweden, including data on the Swedish population, which contains a wide set of variables such as country of birth, education, and mortality.^19^ The cause of death register is held by the National Board of Health and Welfare in Sweden and contains data on the underlying cause of death coded according to the ICD-10 system.

**Figure 1:**
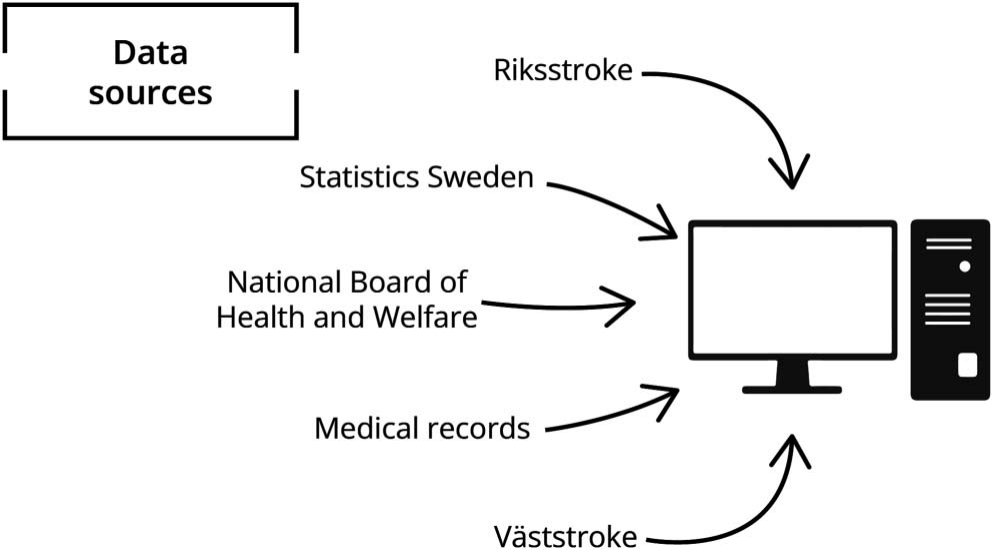
Data Sources

Data on NIHSS at hospital admission (i.e., the first NIHSS on patient presentation at the hospital) were available from both Riksstroke and the Väststroke registers. Because NIHSS was paramount in our study, medical records for patients with no registered NIHSS were reviewed manually by one of the authors to ensure that data on NIHSS were as complete as possible, lowering the number of missing data on NIHSS from 29% to 8%.^20^ See Figure 1 for a visualization of all data sources.

### Variables

To facilitate comparisons in the descriptive analyses, the scale variable NIHSS at admission was both dichotomized (0 points/1 point or more) and categorized (0 points/1 points/2–5 points/6 points or more). Baseline data included the following scale variables: age, systolic blood pressure, and diastolic blood pressure at arrival, and the following dichotomous variables included: sex, stroke type (ischemic/hemorrhagic), comorbidities (antihypertensive medication, atrial fibrillation, diabetes mellitus, previous stroke, previous transient ischemic attack [TIA]), smoking habits, country of birth (Sweden/abroad), education level (low education ≤9 years/higher education ≥10 years), and physical activity before the stroke (inactive/active). The demarcation for being physically active was measured by 2 points or more on the Saltin-Grimby Physical Activity Level Scale, which means that the patient was light to moderately active for at least 4 hours per week.^21^

Our main outcome was a composite measure of death and dependency in activities of daily living (ADL) at 3 months, among previously ADL-independent patients, where a patient who needed help in either clothing or toiletry was considered ADL-dependent. Secondary outcomes, which were covered only in the descriptive analyses, included myocardial infarction during the hospital stay, problems in verbal communication during the hospital stay, diagnosis of swallowing problems, aphasia and dysarthria at discharge, new stroke within 90 days, and total mortality within 90 days including whether the cause of death was from a cerebrovascular disease or for any other reason (ICD-10 codes I60-I69/other reason). Furthermore, the following outcomes were included from the three-month follow-ups: if the patient still experienced any problems from the stroke and if the patient had returned to the same activities as before the stroke, mRS score, and ADL dependency. The latter 2 were collected through questions regarding functional status. Finally, we also included 2 acute care episode measures: if reperfusion therapy was given (including thrombolysis and/or thrombectomy) and the hospital length of stay (days). All included variables were chosen based on availability in the registries, clinical expertise, and discussion among the authors.

### Statistical Methods

Patient characteristics and missing data were analyzed on all patients and are presented as absolute numbers and proportions. Differences between groups of patients depending on NIHSS points at admission were studied using the Pearson χ^2^ test for dichotomous variables and the Mann-Whitney *U* test for scale variables. Univariable (unadjusted) analyses and multivariable (adjusted) analyses on patients having a 0-NIHSS stroke were performed using logistic regression models. The Box-Tidwell test was used to assess whether the scale variables were linearly associated with the outcome. Multicollinearity was checked by variance inflation factor statistics and by creating a bivariate matrix for correlations, where a variance inflation factor value of less than 5 and a Spearman correlation coefficient of less than 0.7 were considered acceptable.

We used multiple imputation on the whole cohort to minimize bias in the unadjusted and adjusted analyses among those with a 0-NIHSS stroke, using both patient characteristics and outcomes as predictors, while only imputing data on patient characteristics. Five imputations with 10 iterations each were conducted using the fully conditional specification method based on a logistic regression model for categorical variables and a linear regression model for scale variables. A sensitivity analysis on complete case data including only cases with complete background data before imputation was also conducted. We did all data handling and statistical analyses in IBM SPSS Statistics and Microsoft Excel. All statistical tests were 2 tailed; the alfa level was set at 5%.

### Standard Protocol Approvals, Registrations, and Patient Consents

Ethical approval for the study was obtained from the Swedish Ethical Review Authority (DNR 346-16 and DNR 2021-03324). Written patient consent was not required because research using quality registers (Riksstroke and Väststroke) is exempt from patient consent according to the Swedish law. However, patients are informed about the possibility to opt out from the registers.

### Data Availability

The data cannot be made publicly available because of Swedish regulations and due to the sensitive nature of the data. However, researchers may apply for the data from Katharina Stibrant Sunnerhagen after obtaining necessary approvals.

## Results

### Exclusion Analysis

**Figure 2:**
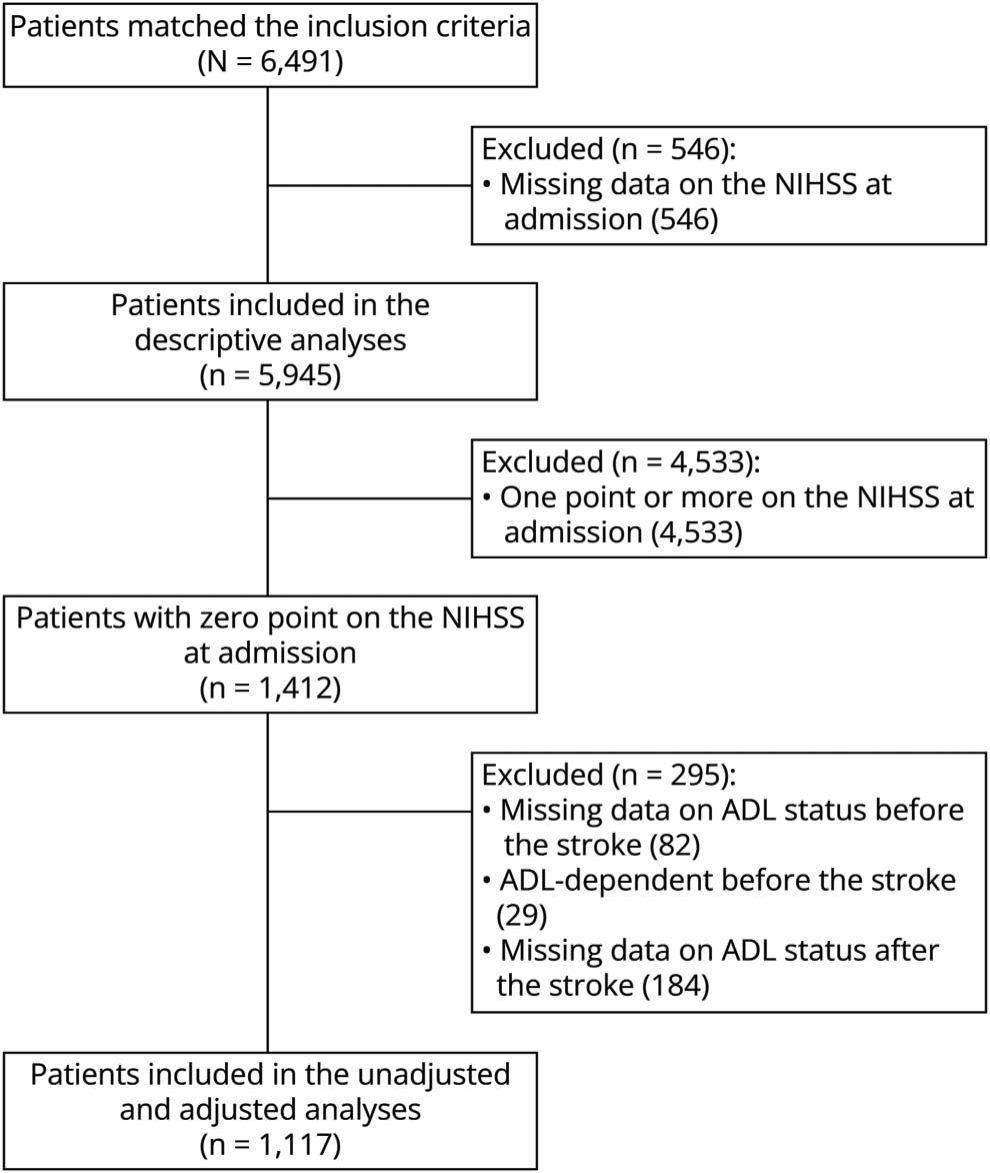
Exclusion Flowchart

A total of 6,491 patients matched the inclusion criteria. At admission, NIHSS was unavailable among 546 (8%) patients, leaving 5,945 for inclusion in the descriptive analyses. Of those, 1,412 had a 0-NIHSS stroke. Of these, 82 had missing data on ADL status, and 29 were ADL-dependent before the stroke, leaving 1301 ADL-independent patients before the stroke. Among these patients, 184 had missing data on ADL status after the stroke, leaving 1,117 patients with a 0-NIHSS stroke for inclusion in the regression analyses (Figure 2).

### Patient Characteristics

Among patients with a 0-NIHSS stroke, 600 (42%) were female patients, the median age was 72 years, 86 (6%) had a hemorrhagic stroke, 220 (16%) had atrial fibrillation, and 1,301 (98%) were ADL-independent before the stroke. In comparison, among patients with 1 point or more on the NIHSS, 2,223 (49%) were female patients, the median age was 77 years, 574 (13%) had a hemorrhagic stroke, 1,411 (32%) had atrial fibrillation, and 3,719 (88%) were ADL-independent before the stroke. Patients presenting with 1 point or more on the NIHSS were also more often physically inactive (61%), compared with those who had a 0-NIHSS stroke (42%). See Table 1 for missing data and comparisons between the groups. We also compared baseline data for more NIHSS levels, 0-NIHSS strokes separately against those with 1 point on the NIHSS, and group comparisons of ischemic stroke vs hemorrhagic stroke and reperfusion therapy vs no reperfusion among patients with 0-NIHSS, see eTables 1–3 (links.lww.com/CPJ/A456).

**Table 1:**
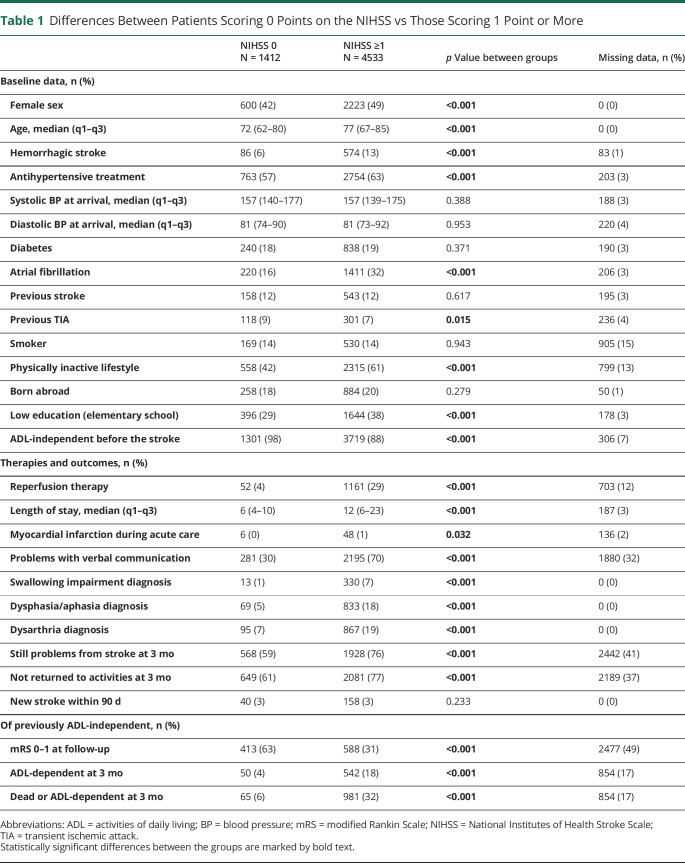
Differences Between Patients Scoring 0 Points on the NIHSS vs Those Scoring 1 Point or More

### Therapies and Outcomes

Reperfusion therapy was given to 52 (4%) patients with a 0-NIHSS stroke, while 1,161 (29%) received reperfusion therapy among patients with 1 point or more on the NIHSS. The median length of hospital stay was also longer among the latter with 12 days, compared with 6 days among 0-NIHSS strokes. Other factors with large differences included problems with verbal communication and swallowing impairment, which were less frequent among those with a 0-NIHSS stroke. Among patients with 1 point or more on the NIHSS, 981 (32%) were either dead or ADL-dependent at 3 months, while 65 (6%) among those with 0-NIHSS strokes had the same unfavorable outcome. Fifteen of these 65 patients were dead, of whom 8 died of cerebrovascular diseases and the remaining 7 patients died of other causes. Another noteworthy outcome is that only 6 (0.4%) patients with a 0-NIHSS stroke and 48 (1%) patients with 1 point or more on the NIHSS had a myocardial infarction during acute care. See Table 1, eTable 1, and eTable 2 (links.lww.com/CPJ/A456) for more details.

### Factors Associated With Death and ADL Dependency at 3 Months

In the unadjusted logistic regression analyses, the following patient characteristics were significantly associated with an increased odds of death and ADL dependency 3 months after a 0-NIHSS stroke: age (odds ratio [OR] 1.06 per year, 95% confidence interval [CI] 1.04–1.09), treatment with antihypertensive drugs (OR 2.07, 95% CI 1.19–3.62), diabetes mellitus (OR 1.90, 95% CI 1.07–3.39), atrial fibrillation (OR 2.07 (95% CI 1.17–3.66), prestroke physical inactivity (OR 3.28, 95% CI 1.92–5.60), and low education level (OR 2.36, 95% CI 1.42–3.92). However, when adjusting for all factors in the multivariable analysis, only age (OR 1.05 per year, 95% CI 1.02–1.08) and physical inactivity (OR 2.48, 95% CI 1.40–4.38) remained significantly associated with an increased odds of death and ADL dependency at 3 months, while low education (OR 1.74, 95% CI 1.00–3.00) remained borderline significant (Table 2). The sensitivity analysis only including cases with complete background data showed similar although slightly attenuated results, with the only major difference being that education no longer were significantly associated with an increased odds of death and ADL dependency (eTable 4, links.lww.com/CPJ/A456).

**Table 2:**
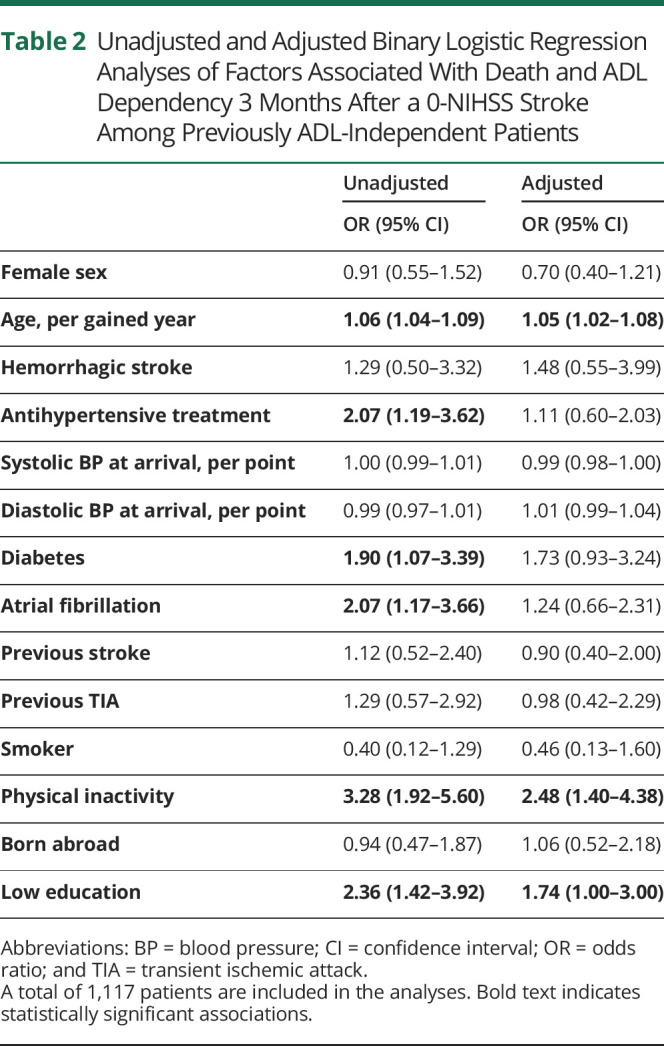
Unadjusted and Adjusted Binary Logistic Regression Analyses of Factors Associated With Death and ADL Dependency 3 Months After a 0-NIHSS Stroke Among Previously ADL-Independent Patients

## Discussion

We found that among previously ADL-independent patients who had a 0-NIHSS stroke, 6% had either died or were ADL-dependent 3 months after the stroke. We also found that old age and prestroke physical inactivity significantly increased the odds of an adverse outcome among these patients.

In total, 24% of the patients in our study had a 0-NIHSS stroke at admission, which contrasts previous studies where incidence rates have ranged from 1% to 4%.^13,16^ The discrepancy in incidence compared with previous studies is not entirely clear but could be attributable to a few different factors. First, the previous studies have included patients between 2003 and 2013.^13,16^ During the same time, stroke severity has decreased, increasing the incidence rates for mild strokes.^22,23^ Second, a 27-month long national stroke awareness campaign was conducted in Sweden between 2011 and 2013, increasing the proportion of patients seeking medical care for their stroke regardless of severity.^23,24^ Third, Swedish health care is tax funded, apart from a minor copayment made by the patient, making it available and affordable for all citizens. This is not the case in the previous studies which were from Switzerland and the United States, where health care instead relies on a health insurance system where patients, although they are covered, often have to pay a certain percent of the total cost by themselves. This makes health care relatively expensive for the individual, which probably make patients with perceived mild symptoms less prone to seek medical attention compared with patients with severe symptoms.

However, when we compare our results with national data in Sweden, 0 point on the NIHSS has been the most common score among all stroke patients since NIHSS was introduced in the annual reports 2012 (before that stroke severity was only measured by the level of consciousness in Riksstroke), with percentages rising from ∼13% of all strokes in 2014 to ∼17% in 2019.^10,25^ At the same time, the missing data levels of admission NIHSS have decreased from 52% to 39%, indicating a bias toward higher NIHSS with more missing data, which probably partly explain the relatively high proportion of 0-NIHSS strokes in our study where the levels of missing data for NIHSS are considerably lower.^10,25^ Comparable national data from Norway show similar proportions as Sweden, with 0-NIHSS strokes accounting for 15% of all strokes in 2020, while data from England and Wales are lower at 7% between 2013 and 2017.^26,27^ Finally, because this is a retrospective study with no standard protocol for all patients, differences in NIHSS status assessment probably differ more than in prospective studies with more rigid assessments. However, all physicians caring for stroke patients in the emergency setting in Gothenburg receive training in how to perform the NIHSS since 2008, which vouches for better quality of the data compared with whether the physicians had not received any training.

Looking at patient characteristics, one of the most prominent differences is that atrial fibrillation was only half as prevalent among patients with a 0-NIHSS stroke compared with those with 1 point or more. This is not surprising because atrial fibrillation is a risk factor for cardioembolic strokes, which in turn are associated with more severe strokes.^28^ Another interesting difference is the gap in prestroke physical activity, where 6 of 10 patients with a 0-NIHSS stroke were physically active, compared with 4 in 10 among those with 1 point or more on the NIHSS. This is also in line with previous studies from the same study population showing that prestroke physical activity reduces stroke severity.^29,30^ The difference in ADL status before the stroke between 0-NIHSS strokes and those with 1 point or more on the NIHSS at admission is also noteworthy. We believe that this is probably because ADL status, just like physical activity, to some extent is a measure of a person's overall health, where those with better health seem to do better in stroke severity.

The finding that the median length of hospital stay is shorter among 0-NIHSS patients compared with those with 1 point or more on the NIHSS was expected because the need for rehabilitation would be less extensive and the risk of in-hospital complications was lower.^31^ The finding that fewer receive reperfusion therapy among patients with 0-NIHSS stroke was also expected because the risk vs reward of thrombolysis among mild strokes is less clear compared with more severe strokes. However, the use of thrombolysis has increased in recent years even among mild strokes, largely after the insight that these strokes also can give rise to disabling symptoms.^32^ Recent findings also show that among mild strokes, patients with both disabling and nondisabling symptoms benefit equally from reperfusion therapy.^33^

The difference in the rates of myocardial infarctions between those with 0-NIHSS strokes and those with 1 point or more on the NIHSS was interesting but not surprising given the variations in patient characteristics between the groups, where the latter were older and more ADL-dependent before the stroke, indicating a less favorable prestroke health status. It was reassuring, however, that only 1% suffered a myocardial infarction during acute care, which is consistent with a study from Austria.^34^ The finding that 1 in 3 reports verbal problems 3 months after the stroke indicates that the NIHSS, although focused on language, is not perfect in detecting all problems. Considering the levels of dysphasia and dysarthria diagnoses at only 5% and 7%, the high levels of patient-reported problems hint that more extensive symptom screening is warranted during the acute care episode.

The only previous study on death and functional outcomes after a 0-NIHSS stroke showed a mortality rate of 4% 1 year after the stroke.^13^ Because we studied mortality at 3 months, our finding of a mortality rate of 2% is not entirely comparable. Regarding functional outcomes, ADL dependency as defined in our study would be equal to at least 4 points on the mRS, and hence, comparisons to the previous study are not possible. However, the patient-reported outcome regarding whether patients still experience problems from the stroke is similar to 1 point or more on the mRS. Our finding that 59% of the patients with a 0-NIHSS stroke experienced problems is slightly more than the previous study which reported that 47% had a mRS score of 1 or more at 3 months.^13^

A major strength of our study is that all stroke units in Gothenburg report to both Riksstroke and the Väststroke register, where the former maintained a coverage (the proportion of stroke patients admitted to hospitals that is also registered in Riksstroke) of 85%–87% during the study period for the included sites. Validation studies have also shown a slight overdiagnosis of stroke in routine care; hence, the true coverage is estimated to be in excess of 90%.^34^ The excellent coverage in combination with the structure of Swedish health care, where access to care is available to every citizen, minimizes selection bias. Other major strengths are that the data are prospectively collected with low levels of missing data for most variables, which together with extensive internal and external validation processes vouches for high-quality data.^35^

As in all observational studies, the choice of variables is limited. To facilitate comparison with previous studies, it would be desirable to compare prestroke mRS with mRS at 3 months. This, however, was not possible because prestroke mRS was unavailable. Other drawbacks are that prestroke physical activity and the variables from the three-month follow-up were patient-reported and not objectively measured. Another limitation is that there was a small discrepancy in patient registrations between Riksstroke and the Väststroke register which gave rise to missing data in some of the background variables for a small proportion of the patients. However, the total level of missing data was low, and the similar results between the imputed data set and the complete case analysis support the accuracy of the findings.

Death is impeccably reported to Statistics Sweden. However, ADL dependency at 3 months had 14% missing data among patients with a 0-NIHSS stroke. We compared the patients who answered the three-month follow-up with those who had missing data on the variable and found that the latter were 6 years younger on average. In addition, they were twice as often born abroad, more often had diabetes, smoked, and were physically inactive. The lower age would decrease the risk of a negative outcome, whereas the sedentary lifestyle would increase the risk; hence, it is hard to predict the direction of possible bias. However, 14% missing data are still relatively acceptable, which in combination with no clear direction of the bias makes us believe that the results are robust. By including all patients in a large area, we believe that our results are generalizable to patients in Sweden and in similar health care organizations abroad.

We found that 6% of the patients were either dead or ADL-dependent 3 months after a 0-NIHSS stroke, highlighting that these strokes are not always benign, and more extensive symptom screening might be needed. The older and physically inactive are at greater risk of an adverse outcome.
